# Best research practices for using the Implicit Association Test

**DOI:** 10.3758/s13428-021-01624-3

**Published:** 2021-09-13

**Authors:** Anthony G. Greenwald, Miguel Brendl, Huajian Cai, Dario Cvencek, John F. Dovidio, Malte Friese, Adam Hahn, Eric Hehman, Wilhelm Hofmann, Sean Hughes, Ian Hussey, Christian Jordan, Teri A. Kirby, Calvin K. Lai, Jonas W. B. Lang, Kristen P. Lindgren, Dominika Maison, Brian D. Ostafin, James R. Rae, Kate A. Ratliff, Adriaan Spruyt, Reinout W. Wiers

**Affiliations:** 1 University of Washington, Seattle, WA 98195–1525 USA; 2 University of Basel, Basel, Switzerland; 3 Chinese Academy of Sciences, Beijing, China; 4 Yale University, New Haven, CT USA; 5 Saarland University, Saarbrucken, Germany; 6 University of Koeln, Cologne, Germany; 7 McGill University, Montreal, Canada; 8 Ruhr University Bochum, Bochum, Germany; 9 Ghent University, Ghent, Belgium; 10 Wilfrid Laurier University, Waterloo, Canada; 11 University of Exeter, Exeter, UK; 12 Washington University in St. Louis, St. Louis, MO USA; 13 University of Warsaw, Warszawa, Poland; 14 University of Groningen, Groningen, Netherlands; 15 University of Massachusetts, Amherst, MA USA; 16 University of Florida, Gainesville, FL USA; 17 University of Amsterdam, Amsterdam, Netherlands

**Keywords:** Implicit Association Test, recommended research practices, indirect attitude measurement, implicit social cognition

## Abstract

Interest in unintended discrimination that can result from implicit attitudes and stereotypes (implicit biases) has stimulated many research investigations. Much of this research has used the Implicit Association Test (IAT) to measure association strengths that are presumed to underlie implicit biases. It had been more than a decade since the last published treatment of recommended best practices for research using IAT measures. After an initial draft by the first author, and continuing through three subsequent drafts, the 22 authors and 14 commenters contributed extensively to refining the selection and description of recommendation-worthy research practices. Individual judgments of agreement or disagreement were provided by 29 of the 36 authors and commenters. Of the 21 recommended practices for conducting research with IAT measures presented in this article, all but two were endorsed by 90% or more of those who felt knowledgeable enough to express agreement or disagreement; only 4% of the totality of judgments expressed disagreement. For two practices that were retained despite more than two judgments of disagreement (four for one, five for the other), the bases for those disagreements are described in presenting the recommendations. The article additionally provides recommendations for how to report procedures of IAT measures in empirical articles.

## Introduction

Greenwald and Banaji (1995) reviewed methods and findings in an area of research that they identified as *implicit social cognition*. Their review focused on research by social and personality psychologists—and more specifically on research using *indirect* measures of attitudes, stereotypes, and self-esteem. Their concluding sentence was: “Perhaps the most significant remaining challenge is to adapt these [indirect measurement] methods for efficient assessment of individual differences in implicit social cognition.”

Greenwald et al. (1998) addressed that challenge in an article titled “Measuring individual differences in implicit cognition: The Implicit Association Test”. Their article described three experiments using a method they named *Implicit Association Test* (IAT) to measure attitudes (associations of concepts with valence) indirectly. The subsequent body of reports of research using the IAT as research procedure now exceeds 3000 peer-reviewed articles.^1^

### Definition of ‘implicit’

*Implicit* often appears in psychological publications as an adjective preceding *memory*, *attitude*, *stereotype*, *self-esteem*, *identity*, or *association*. These adjective–noun pairs are often contrasted with pairs in which *explicit* is the preceding adjective. The implicit–explicit contrast has been understood in two ways. Understanding 1 treats *implicit* and *explicit* as properties of psychological *measures*, describing measures that assess a construct indirectly (implicitly) versus directly (explicitly). Understanding 2 treats *implicit* and *explicit* as properties of *mental processes* or *mental representations*, which may be conceived as operating in automatic or unconscious fashion (implicitly) or in controlled or conscious fashion (explicitly).

The mental process/representation understanding derives from memory studies of the 1980s, many of which used indirect measures to reveal operations of memory that occurred without conscious recollection of the memory-creating events (cf. Richardson–Klavehn & Bjork, 1988). By the early 1990s, however, two influential methodological articles (Jacoby, 1991; Reingold & Merikle, 1988) had offered convincing (and subsequently unrefuted) arguments that it was not justifiable either (a) to treat indirect measures as pure indicators of unconscious process, or (b) to treat direct measures as pure indicators of conscious process.

Reviewing the history that preceded their 1995 article that extended the implicit domain to social cognition, Greenwald and Banaji (2017, pp. 861–863) similarly concluded that ‘implicit’ and ‘explicit’ are most justifiably used to describe (respectively) measures that reveal psychological constructs indirectly and directly rather than as synonyms for ‘unconscious’ vs. ‘conscious’.^2^ In introducing the Implicit Association Test, Greenwald et al. (1998) used ‘implicit’ to describe a property of the method they introduced rather than of the construct it was measuring. In a later overview of the research area of implicit social cognition, Fazio and Olson (2003) even more strongly emphasized indirect measurement as the distinctive property of implicit measures.

The most forceful argument for Understanding 2 (i.e., mental process or representation interpretations of implicit and explicit) is that of De Houwer et al. (2009a), who wrote: “the term *implicit* can best be understood as being synonymous with the term *automatic*” (p. 350). (Commentary on their view is available in Gawronski et al., 2009; Nosek & Greenwald, 2009; and in the reply to those by De Houwer et al., 2009b.) A virtue of the presently recommended measurement-based definition (implicit = indirectly measured) is that researchers can readily agree on distinguishing between direct and indirect measures, while it appears more difficult to establish agreement on the extent to which a measure taps automatic versus controlled mental operations. We conclude this discussion of controversy about definition with the (hopefully comforting) observation that this article was easily written so that the differences among readers’ preferred understandings of ‘implicit’ should not affect interpretation or application of the article’s conclusions about recommended research practices.

### Measurement characteristics of IAT measures

References to IAT measures in the remainder of this article refer to the standard form of the IAT, which has seven sets (blocks) of *trials*, each of which presents a stimulus (*exemplar*) belonging to one of the IAT’s two *target* categories or to one of its two *attribute* categories. (This standard procedure is described more fully in Appendix A.) Four of the seven blocks (ordinally, nos. 3, 4, 6, and 7) present *combined tasks* in which exemplars of one pair of categories appear on all odd-numbered trials, and exemplars of the other pair appear on all even-numbered trials. This procedure produces an *indirect* measure (presumably of association strengths), meaning that the subject is given no instruction to report (directly) on association strengths (or attitudes or stereotypes, etc.). The subject’s only instructed task is to press a left key or a right key to classify each exemplar into its proper category. The same two response keys are used to classify target and attribute concepts, with correct response sides for the two target categories being switched (from those used initially in Blocks 3 and 4) between right and left for the second combined task (in Blocks 6 and 7). The implicit measure is determined mainly by the latency difference between these two combined tasks, which provides the numerator of the IAT’s *D* measure, for which the now-standard scoring algorithm is described in Appendix B.

For IAT measures, the psychometric properties of greatest interest are (a) interpretation of the IAT’s zero point, and (b) its statistical reliability. Greater detail on these properties than is presented here can be found in Greenwald et al. (2020).

### Interpretation of the IAT’s zero point

The initial publication of the IAT described it as a measure of “differential association of two target concepts with an attribute” (Greenwald et al., 1998, p. 1464). As described in Appendix B, the numerator of the IAT measure is computed as the difference in average response speed between the IAT’s two combined tasks. Greenwald et al. (1998) interpreted the zero value of their race attitude IAT measure—obtained when the race attitude IAT’s two combined tasks were performed at equal average speed—as indicating absence of preference between racial Black and racial White (p. 1476). This interpretation is important, because it justifies interpreting values deviating from zero in one direction (numerically positive, as used by most researchers) as indicating automatic preference for White race, and values deviating from zero in the negative direction as indicating automatic preference for Black race.^3^

The absence-of-preference interpretation of an attitude IAT’s zero point has been debated in several publications (Blanton & Jaccard, 2006; Blanton et al., 2015; Cvencek et al., 2020; Greenwald et al., 2006). In their 2015 article, Blanton et al. offered a regression method to argue that the absence-of-preference point for race attitude measures should be properly located at a numeric value of the IAT score approximately 0.5 SD higher (numerically, more positive) than the standard scoring algorithm’s zero-point. That conclusion led to their claim that IAT measures substantially overestimate (by about 30%) the proportion of any research sample (or population that the sample represents) that merits characterization as showing implicit white racial preference.

A method for validating the absence-of-preference interpretation of the IAT’s zero point is available using balanced identity theory (BIT; Greenwald et al., 2002). Predictions from BIT’s *balance–congruity principle* describe properties of trios of association-strength measures that should not be empirically confirmable unless the zero points of all of those association-strength measures validly indicate absence of difference in strengths of complementary associations. Data confirming those BIT predictions, and thereby validating the absence-of-difference interpretation of the IAT’s zero point, were reported by Cvencek et al. (2020; see their Fig. 3 and its accompanying text). Cvencek et al.’s meta-analysis of 36 studies included data from 9808 IAT respondents and used a variety of trios of IAT measures. Their most compelling empirical test, derived from BIT’s balance–congruity principle, was the non-obvious prediction that, in a collection of balanced identity studies using trios of IAT measures, the linear regression of measures of correlation between two of the three measures in a balanced identity study on the means of the study’s third measure (a) should be positive in slope and (b) should pass through the origin of the regression’s scatterplot (Greenwald et al., 2002, pp. 9–10). The meta-analysis of 36 studies, each with three IAT measures, permitted constructing a regression scatterplot with 108 (= 36 × 3) data points, each obtained from the correlation of the mean of an IAT measure with the correlation between two other IAT measures. The trios of variables in these studies varied widely in content, allowing a test with much more power and precision than was available in a previous review (of 18 studies) by Cvencek et al. (2012). The predicted positive regression slope was found very clearly (*r* = .84). More importantly for the zero-point test, the intercept of the scatterplot of this regression passed almost exactly through the scatterplot’s origin. The intercept, estimated in units of the IAT’s D measure, was – .005 (95% CI = – 0.048 to 0.038).^4^

### Test–retest reliabilities and internal consistencies of IAT measures

Greenwald and Lai’s (2020) review of implicit social cognition included an original meta-analysis, from which they reported that test–retest reliabilities for IAT measures averaged *r* = .50 (data from 58 studies) and that internal consistencies of IAT measures averaged α = .80 (data from 257 studies).

The greater value for internal consistency than for test–retest reliability indicates the presence of systematic variance in single IAT observations that is not shared across measurement occasions. More specifically, the aggregate α = .80 indicates that the IAT captures 80% of the systematic (i.e., non-noise) variance in whatever combination of latent variables underlies a single-occasion measure. Likewise, interpretation of aggregate test–retest reliability of .50 is that 50% of the single-occasion measure’s variance represents a latent variable that is stable across measurement occasions (with the other 50% being variable across measurement occasions).^5^

The moderate reliability of IAT measures is, unfortunately, often reduced by requirements of non-standard research situations. When researchers have limited data collection time, as is often true of Internet data collections, test–retest reliability may be reduced because of researchers opting to save time by reducing numbers of IAT trials below the numbers recommended in Appendix A. Examples of such reductions are Study 4 reported by Lai et al. (2014) and all studies reported by Lai et al. (2016). Limited attention span of young children similarly obliges reduced numbers of IAT data collection trials (e.g., Cvencek et al., 2011).

The IAT’s average test–retest reliability of .50 is adequate for studies that assess correlations of IAT measures with other measures or for studies that test hypotheses about group differences or experimental treatment differences in mean IAT scores. However, test–retest reliability of *r* = .50 is not adequate to justify treating a single IAT observation as accurately diagnostic of an individual’s attitude, stereotype, identity, or self-esteem. Notwithstanding, one can create an IAT measure that is adequately diagnostic for single persons by repeating IAT measurements, much as is routinely done in research using blood pressure measures. Blood pressure measures, when used in research on hypertension, are routinely repeated two, three, or more times on the same person, within a research session, to provide a measure that is adequately diagnostic for each research subject (see Smith, 2014; Stergiou et al., 2002). As an example of the prospects for improving reliability of IAT measures by averaging multiple observations, data from Lindgren et al. (2018) were used (by Greenwald et al., 2020) to show that the average of eight IAT measures obtained from single subjects over a 2-year period had test–retest reliability of *r* = .89, a level more than sufficient for an IAT score to be treated as diagnostic for a single person.

Greenwald and Lai’s (2020) meta-analysis of reliability of IAT measures found that test–retest reliabilities obtained at the longest intervals in their data set were similar in magnitude to those obtained with intervals between 0 and 2 days. Overall, what is known about test–retest reliability and internal consistency of IAT measures agrees with the conclusion from Connor and Evers’s (2020) methodological study that IAT measures capture stable properties of persons, albeit in statistically noisy fashion.

## Best Research Practices

Three subsections will describe, first, recommended practices for selecting categories and exemplar stimuli for IAT measures; second, recommended practices for administering IAT measures; and third, recommended practices for reporting procedures and results obtained with IAT measures. These recommended practices developed from a larger set that was described in a working manuscript that the first author circulated to multiple colleagues in September of 2018. Comments by these experts led to refinements through three subsequent manuscript drafts. This article’s Postscript more fully describes this 7-month refinement process.

The listing of recommended practices does not include two important practices that are described in this article’s appendixes. Appendix A describes what is widely recognized as the standard procedure for presenting an IAT measure. Appendix B describes a scoring procedure for IAT measures that has been treated as a standard since its publication (Greenwald et al., 2003). The two appendixes also describe acceptable variations in these standard procedures.

### Best practices for selection of categories and exemplar stimuli for use in IAT measures

The following recommendations for practices A1–A9 and B1–B12 (Table 1) have two sources: (a) published experimental studies of procedural variations and (b) informal knowledge accumulated in years of research experience by the present set of authors and others. Most of the experience-based recommendations have been learned from pilot testing of novel IATs (as recommended in A8, below). For some of the 21 practices, this article’s description is the only published description. The authors (sometimes not in total unanimity) regard each of the practices that has not yet been tested experimentally as having a plausible rationale that supplements the informal evidence of usefulness obtained in pilot testing. Those interested in varying from these not-yet-experimentally evaluated procedures are encouraged to include them in experimental tests alongside suspected comparable or superior procedures.

**Table 1:** Recommended practices for selecting categories and exemplar stimuli, and for administering IAT measures

A. Best Practices for Selection of Categories and Exemplar Stimuli for Use in IAT measures
A1. All four categories used in the IAT should be familiar to subjects
A2. The primary criterion for selection of exemplar stimuli for each target and attribute category is that they must be *easy* for subjects to sort correctly
A3. Exemplars for any target category should differ from those for its contrasted target category in just *one* primary feature or *one set* of highly correlated features; the same should be true for exemplars of the two attribute categories
A4. For IATs designed to measure stereotypes, avoid confounding the stereotype’s contrasted attributes with valence
A5. Avoid exemplars for one attribute category that are negations of possible exemplars for the contrasted attribute category
A6. Negations can be satisfactory in category labels
A7. In selecting attribute exemplars, avoid exemplars that have an idiosyncratic additional basis for association with either of the two target concepts
A8. Exemplar stimuli for target and attribute categories are best selected by pilot testing using the category classification tasks planned for the IAT
A9. When all four concepts in an IAT are expressed as words, font variations can be used to help subjects distinguish target exemplars from attribute exemplars
B. Best Practices for IAT Administration Procedures
B1. Counterbalancing the temporal order of the two combined tasks is generally desirable
B2. Counterbalancing of sides initially assigned to each category is desirable
B3. Target and attribute category trials are *always* strictly alternated in the standard IAT's procedure for combined-task blocks
B4. Intertrial intervals should be brief
B5. Initial practice in classifying the two target concepts (first block) should precede initial practice in classifying the two attribute concepts (second block)
B6. It is desirable to use at least 3 exemplars for each category in the IAT
B7. It is desirable (not essential) for the number of trials in any block to allow each target exemplar stimulus to be presented the same number of times within the block, and likewise for the exemplars in each attribute category
B8. Runs of more than four consecutive same-key-correct trials in combined-task blocks are undesirable
B9. In correlational studies, statistical power can be increased by using 2 or more administrations of the IAT for each subject
B10. In studies that assess correlations of an IAT measure with other variables, it is desirable for the subject population to display substantial variability in the IAT measure
B11. In laboratory research, when IAT-including experiments are administered by multiple experimenters, treatment conditions should be distributed equally across experimenters
B12. Desirable procedures for pretest–posttest IAT administrations

#### A1. All four categories used in the IAT should be familiar to subjects

Unfamiliar categories cannot be expected to have associations measurable using the IAT. Because of expectable slow classification in responding to exemplars of unfamiliar categories, those categories will appear to be weakly associated with other categories. If exemplars for one of two categories in an attitude IAT are totally unfamiliar, that category will inappropriately appear to be negatively evaluated (as was first found by Brendl et al., 2001). For such categories, an interpretation in terms of the IAT as a measure of association strengths involving the unfamiliar categories is not appropriate. To state this recommendation as simply as possible, the IAT should not be used to measure associations involving unfamiliar categories. However, exemplars for a familiar category need not themselves be highly familiar, so long as they are easily sorted into their assigned categories (see Recommendation A2). This was established experimentally in early IAT studies by Rudman et al. (1999) and by Dasgupta et al. (2000).

The prohibition on totally unfamiliar categories does not preclude conducting experiments using novel categories for which both category labels and exemplars are previously unfamiliar (e.g., the Pokemon characters used by Olson & Fazio, 2001, and the fictitious Niffians and Laapians used by Ranganath & Nosek, 2008). However, studies involving such unfamiliar categories require preliminary training involving the novel categories and their exemplars to make them sufficiently familiar to subjects when the IAT is administered.

#### A2. The primary criterion for selection of exemplar stimuli for each target and attribute category is that they must be *easy* for subjects to sort correctly

Exemplar stimuli that are difficult to categorize will be responded to slowly in the IAT. As in the case of unfamiliar categories (see A1), this slowness can inappropriately cause the category containing those exemplars to appear to be weakly associated with another (target or attribute) category in the IAT. If exemplars for only one of two target categories in an attitude IAT are difficult to categorize, that category may inappropriately appear to be negatively evaluated. If exemplars for both target categories are difficult to classify, there may appear to be no attitude when an attitude might have been detected with use of easily classified exemplars.

An important contributor to easy classification of exemplars is for those exemplars to be *representative* of their categories. Several empirical findings have shown that non-representative exemplars of categories will produce results different from those obtained with representative exemplars (e.g., Bluemke & Friese, 2006; Govan & Williams, 2004; Steffens & Plewe, 2001). For example, Govan and Williams used *nettles*, *skunkweed*, and *poison ivy* as exemplars for the category *flowers*, and used *butterfly*, *grasshopper*, and *firefly* as exemplars for the category *insects*. Recommendation A8 provides a simple method for selecting exemplar stimuli that can be easily classified into their respective categories. Recommendation B6 considers the numbers of exemplars that should be selected.

#### A3. Exemplars for any target category should differ from those for its contrasted target category in just *one* primary feature or *one set* of highly correlated features; the same should be true for exemplars of the two attribute categories

When this practice is followed, subjects can have only one basis for distinguishing exemplars of the two contrasted categories. Consider some examples of violation of this practice: If a (racial) contrast between Asian and African involves only male exemplars of Asian persons and only female exemplars of African persons, subjects can use either gender or race as the basis for distinguishing the two sets of exemplars (see Mitchell et al., 2003, for studies with such multiple categorization possibilities). Or, if words for a positive valence attribute are all shown in green font while those for negative valence are all in red font, subjects are free to sort based on font color rather than valence. In an attitude IAT using the race and valence exemplar contrasts just described, the IAT measure might indicate (depending on subjects’ choice among the available sorting strategies), difference in valence associations of the race groups, difference in valence association of the gender groups, or differences in associations of the font colors with the race or gender groups. Obtaining an interpretable measure requires selecting exemplars that do not allow subjects such flexibility in sorting strategy.

When target concepts are represented by face images, this recommendation obliges consideration of the expressions on those faces. Having smiling faces for one political candidate and frowning faces for another in a political attitude IAT is obviously undesirable. One solution is to have all face images lack facial expression, although this may be difficult to achieve when drawing on available collections of face photos. Equally satisfactory is to use faces with a mixture of smiling and frowning expressions. Matching the frequencies of expressions for the two face categories will deny subjects the opportunity to use facial expression as an alternative guide to classification.

#### A4. For IATs designed to measure stereotypes, avoid confounding the stereotype’s contrasted attributes with valence

Some published IAT studies have assessed stereotypes using trait attribute contrasts that confounded the contrasted traits with valence (examples: strong vs. weak, smart vs. dumb, sober vs. drunk). Such studies are sometimes intercepted on the path to publication by reviewers or editors who will note that the trait contrast was confounded with a valence contrast and the IAT might therefore have provided an attitude measure rather than the desired stereotype measure (cf. Wittenbrink et al., 1997). This confounding would be a deviation from Recommendation A3, by allowing subjects the option of treating the attribute contrast as one of valence, effectively converting the intended stereotype measure into an attitude measure. This problem can often be avoided by selecting contrasted trait categories that do not differ in valence. Rudman et al. (2001) used two solutions for this problem in trying to measure a male=strong stereotype. One solution selected exemplars for strong and weak that were matched in valence. The other, which was considerably easier to implement, was to contrast the attribute presumed to be characteristic of one group (e.g., strength, expected to be more associated with male) with a similarly valenced characteristic of the complementary group (e.g., warmth, expected to be more associated with female). The second strategy simultaneously measured two stereotypes (male=strong and female=warm), which might or might not be desirable, depending on the aims of the research.

The strategy of matching valence for positive and negative stereotyped attributes is challenging enough so that it can be informative to illustrate how this is possible, by describing how it was done by Rudman et al. (2001) in a study measuring differential association of male vs. female with strong vs. weak. The challenge is created by the fact that valence of words meaning *strong* is substantially more positive than valence of words meaning *weak*. In their Experiment 2 matched valence was achieve by using a mixture of (a) negative-valence exemplars for both strong (e.g., fight, fury, violent) and weak (feeble, scrawny, lame), (b) neutral-valence exemplars for both (e.g., durable, loud, oak vs. fragile, quiet, feather) and (c) positive-valence exemplars for both (e.g., bold, mighty, power vs. delicate, flower, gentle).

#### A5. Avoid exemplars for one attribute category that are negations of possible exemplars for the contrasted attribute category

Negations have the attractive feature of being easy to produce. However, as demonstrated by Phelan and Rudman (2008); see also Verschuere & Kleinberg, 2017) negations can cause difficulty in IATs, likely because of an extra processing demand of requiring comprehension of the non-negated meaning before apprehending the negated meaning (see Gilbert, 1991, esp. p. 7). For example, processing ‘unhappy’ requires activating and then negating the meaning of ‘happy’. Some other examples: *trust* and *distrust*, *healthy* and *unhealthy*, *true* and *not true*. The negations in these pairs can be avoided by using instead a synonym (of the negation) that is not in negation form—e.g.: *suspicion* in place of *distrust*, *sick* in place of *unhealthy*, and *false* in place of *not true*.

#### A6. Negations can be satisfactory in category labels

A negation used in a category label must be processed only once in a block of trials, prior to the start of the block, rather than at the time of exemplar presentations. Usefulness of negations in category labels is fortunate because there are categories for which it is impossible to find a good label other than in negation form. An example was a study of smoking-related attitudes by Swanson et al. (2001). The exemplars for the category *smoking* were pictures containing cigarettes. The contrasted category’s exemplars were the same scenes, with the cigarettes omitted. For this contrasted category, it was not possible to find a better label than *non-smoking*. Many studies have successfully used *Me* vs. *Not me* as category labels in self–concept or self–esteem IATs, as an alternative to *self* vs. *other* (see Greenwald & Farnham, 2000) . The *Me* vs. *Not-me* contrast is especially useful when conducting self-related IATs with young children, for whom the contrast of *self* vs. *other* as category labels may pose a comprehension challenge (see Cvencek et al., 2011).

#### A7. In selecting attribute exemplars, avoid exemplars that have an idiosyncratic additional basis for association with either of the two target concepts

Some otherwise acceptable attribute exemplars may be compromised by strong association with one of the target concepts in the same IAT. Such problems occur infrequently and, fortunately, they also tend to be obvious. One such example is selecting *cancer* as a negative valence exemplar in an IAT designed to measure attitude toward smoking—the problem is due to *cancer* being associated with the target concept of *smoking* (and not with *non-smoking*) through its association with *health* rather than (or in addition to) its valence.

This recommendation was one of two (the other was B1) for which more than 2 of the 29 persons who evaluated the recommendation indicated a disagree judgment. (See this article’s Postscript for description of how judgments of agreement and disagreement were obtained.) The four objectors to A7 offered similar reasoning—that this recommendation would exclude an attribute exemplar that could be effective when used in an IAT measure. A reasoned argument opposing that objection (given in the next paragraph) was not included in the draft that the evaluators initially responded to.

Assume that, counter to Recommendation A7, “cancer” is used as a negative valence exemplar in an attitude IAT contrasting target concepts of physical illness vs. mental illness. In the combined task that assigns the same key to negative valence and mental illness, cancer’s association with physical illness will tend to elicit an incorrect response, slowing average latency. In the other combined task, which assigns the same key to physical illness and negative valence, cancer’s association with physical illness will facilitate a correct response. The net effect, which is a biasing of the measure toward greater negativity of physical illness (for all subjects), can and should be avoided by not using ‘cancer’ as an exemplar of negative valence in this IAT.

#### A8. Exemplar stimuli for target and attribute categories are best selected by pilot testing using the category classification tasks planned for the IAT

This recommendation follows on the earlier point (A2) about ease of classification being a requirement in selecting category exemplars. Subjects for pilot testing should come from the intended research subject population. The designer of any IAT is often the first pilot subject, which is entirely satisfactory and appropriate if the IAT designer is representative of the planned subject population. A judgment as to whether specific exemplars are easy enough to classify can be based on examination of data obtained from pilot subjects. The useful data will come from Blocks 1 and 2 of the standard procedure (see Appendix A). Pilot subjects should be able to categorize all stimuli in these two blocks rapidly (average latency in the range of 600–800 ms for most young adult subjects) and with low error rates (less than 10%).

Exemplars that just one of a small group of pilot subjects finds difficult to classify are safely discarded without further consideration. There is no need for selection criteria such as word length, word frequency, or meaningfulness, even though these criteria are appropriate for many other investigations of categorization. An obvious exception to this just-stated observation is that word characteristics should not be confounded with a category contrast, such as by using short words as exemplars for one category and long words as exemplars for its contrasted category; this would be a deviation from Recommendation A3.

#### A9. When all four concepts in an IAT are expressed as words, font variations can be used to help subjects distinguish target exemplars from attribute exemplars

In the very first published IAT (an attitude measure that contrasted target concepts of flowers versus insects), all four categories were presented as lowercase words. Some subjects in that experiment pointed out that they were sometimes uncertain whether a target concept’s exemplars (e.g., lily or rose) were to be sorted as *flower* (target concept) or as *pleasant* (attribute concept). Likewise, maggot and roach might be classified as *insect* (target concept) or as *unpleasant* (attribute concept). To avoid or reduce this difficulty for subjects, a case variation was introduced in the second and third experiments of that first IAT report (Greenwald et al., 1998). Valenced attribute exemplars were displayed in all lowercase and target concept exemplars were displayed with initial capital letters. More substantial font differences between attribute and target concept exemplars are not problematic. The target–attribute distinction can be enhanced by simultaneously varying font color (e.g., green vs. blue), case (upper vs. lower), and typeface (e.g., Courier vs. Arial) between target and attribute exemplars.

### B. Best practices for IAT administration procedures

Likely because of the IAT’s frequent appearance in empirical reports, authors have become content with minimally reporting details of IAT administration procedures. There is often not even the citation of a publication in which a reported IAT’s procedures were previously described in detail. Consequently, if sub-standard procedures were used, this may not be detectable in a published report. An opposing force is that the culture of the field is to share procedures among researchers, with some likelihood that the most widely shared procedures are ones developed by researchers who incorporated presently recommended practices. If the most widely shared procedures incorporate strong administration procedures, the level of non-reporting of procedural details may not be a concerning problem. However, it is a problem that many editors do not insist on reporting of at least the most important aspects of procedures. This section on IAT administration procedures is therefore followed by a section describing components of procedure that should be most useful to describe in published reports.

#### B1. Counterbalancing the temporal order of the two combined tasks is generally desirable

With two target categories (call them T1 and T2) and two attribute categories (A1 and A2), the first combined task can assign the same key to T1 and to either A1 or A2 (and T2 to the same key as the other attribute category). The earliest IAT studies observed an order effect such that the association of T1 with A1 (and T2 with A2) was significantly stronger when T1 and A1 were assigned to the same key in the first combined task rather than when they were assigned to the same key in the second combined task. To avoid having this effect of combined-task order on an IAT measure influence observed means of IAT measures within experimental treatments, it is desirable to counterbalance, across subjects and within treatment conditions, the order of administration of the two combined tasks.

This recommendation was the second of the two that elicited judgments of disagreement from more than two of the 29 evaluators. The five objectors all assumed that it should be desirable to avoid an (undesired) effect of the counterbalancing on correlations of IAT measures with other variables of interest. More specifically, the objectors’ expectation was that the extraneous variance produced by counterbalancing would reduce correlations between IAT measures and other measures of interest.

The goal of maximizing correlations is reasonable, but the assumption that use of this (or other) counterbalancing will obscure correlations has two problems. First, researchers can correct for possible reductions of correlation by using the counterbalanced variable as a covariate to adjust observed correlations among other variables. Second, the effect of order of combined tasks is typically small, meaning (statistically) that its effect on correlations with other variables is likely to be very small, possibly unnoticeable. 6 The gain from using the counterbalancing is that sample means will not be displaced due to the chosen order of combined tasks influencing the mean IAT value for the entire sample.

#### B2. Counterbalancing of sides initially assigned to each category is desirable

There have been no empirically demonstrated effects on IAT measures of either (a) the attribute category initially assigned to the left or right key or (b) the side to which each target concept is initially assigned. Nevertheless, some researchers suspect that positioning the positive valence category on the right side may produce a small effect of faster responding than if negative valence is assigned to the right key.^7^ This counterbalancing is relatively easy to achieve and is especially desirable in studies with large respondent samples, in which small effects may prove statistically significant. Note that the basis for counterbalancing here is the general principle that arbitrary procedural variations are best distributed randomly across subjects and treatments, which also applies to the order variation considered in B1. For B1, the known effect of order on IAT scores definitely strengthens the justification for counterbalancing, which is why Recommendation B2 is stated as “desirable” without the added strengthening (“generally desirable”) used for B1.

#### B3. Target and attribute category trials are *always* strictly alternated in the standard IAT’s procedure for combined-task blocks

The desirability of the strict alternation procedure was discovered informally (and repeatedly) in variations of IAT procedures tested in 1994–1995 by the authors of the first IAT publication. The main supporting evidence was that measured IAT effects consistently had larger effect sizes when this procedure was used. The authors regarded this strict alternation procedure as important enough to mention it in five places in that initial publication (Greenwald et al., 1998). Maximizing task switches between target concept and attribute concept classification should have effects of both increasing facilitation of IAT performance in one IAT combined task and interfering in the other combined task. Most published reports of IAT measures presumably use this standard alternation, although use of this procedure is rarely mentioned in publications. Occasional reports do mention deviating from the strict alternation for a specific research purpose (e.g., Mierke & Klauer, 2003; Rothermund et al., 2009). No published report has yet indicated that deviation from strict alternation improves either the IAT’s psychometrics or its correlation with conceptually related measures.

This article’s recommendation is to use the standard alternation between target and attribute discriminations in combined tasks blocks. Readers may assume, if no mention is made of this procedure, that researchers used the standard alternation strategy in combined task trial blocks, but it would be better to describe that explicitly.

#### B4. Intertrial intervals should be brief

Greenwald et al. (1998) varied the interval between initiation of a response on Trial *n* and presentation of the stimulus for Trial *n*+1 among values of 100, 400, and 700 ms. They found no effect of this variation on magnitude of effects obtained with IAT measures. After that early finding, researchers have tended to use quite brief intertrial intervals (250 ms is a commonly used value). This conserves time in a procedure that often has a few hundred trials. (For the standard 190-trial IAT procedure described in Appendix A, adding 1 second to the intertrial interval will increase the procedure’s duration by about 3 min.) A suspected additional virtue of the brief intertrial interval—albeit one not studied systematically—is to limit intertrial time that can be used to allow mental rehearsal of the correct response key assignments. Greater intertrial time would plausibly reduce difficulty in combined tasks that assign the same key to two non-associated concepts; such opportunity to rehearse instructions between trials may permit faster responding, which might in turn reduce the IAT’s sensitivity to differences in association strengths.

#### B5. Initial practice in classifying the two target concepts (first block) should precede initial practice in classifying the two attribute concepts (second block)

This conclusion was drawn from never-published exploratory studies conducted prior to the first IAT publication. The explanation: When attribute concept practice comes first, the attribute initially assigned to the left key can acquire some association with that key. The ensuing initial practice classification of the target categories may then increase the association between the target concept practiced on the left key in the second block and the attribute previously practiced on the left key. The psychological principle underlying this recommendation is *mediated generalization* (Cofer & Foley Jr., 1942), a process by which two categories (e.g., *pleasant* and *insect*), both associated with the same response (e.g., *left key*), can thereby become associated with each other. In the recommended procedure, when target concepts are practiced first, *left key* may acquire an association with *insect* in the first bock. In the second block, *insect* may gain some association to *pleasant* by mediated generalization (due to their sharing the left key during the two practice blocks). In the non-recommended procedure of practicing attribute classification first, *pleasant* acquires association with *left key* in the first block; then, due to mediated generalization, *insect* gains some association with *pleasant* in the second block. Despite the conceivable operation of mediated generalization regardless of order of administering the first two blocks, there is a theoretically expected asymmetry of the two orders. In the second block, the direction of association formation should be from the category practiced in the second block to the one practiced on the same key in the first block. The expected stronger effect of practicing *insect* in the second block is that it is easier to form the insect-to-pleasant association than the pleasant-to-insect association. This asymmetry is explained by Paivio’s (1969) *conceptual peg* hypothesis, based on experiments showing stronger acquisition of associations in noun–adjective (i.e., target–attribute) direction than in adjective–noun (i.e., attribute–target) direction.^8^

#### B6. It is desirable to use at least three exemplars for each category in the IAT

In the only experimental study that varied number of exemplars for IAT categories, Nosek et al. (2005) found that as few as two exemplars could be used to represent categories of pleasant, unpleasant, young, old, male, female, science, and liberal arts. Use of a single item (the category label) for each category did not fail totally, but was clearly inferior. These results should be generalized cautiously because of the limited number of categories and IAT measures investigated. This caution is applied here in recommending a minimum of three items per category. In published studies using the IAT, the numbers of exemplars per category are mostly in the range of four to six. Using four or more exemplars should minimize risk that the category’s effective definition in the IAT may be distorted by the specific exemplars chosen.

From another perspective, some authors have recommended using two or more interchangeable sets of exemplars for categories when it is easy to generate sufficient numbers of easily classifiable exemplars (as it is for categories such as positive/negative valence, male/female gender, young/old age, and black/white race (and many others). Wolsiefer et al. (2017) analyzed the effects of exemplar choice in IAT measurement. They found that variation due to use of different sets of exemplars was smaller in IAT measures than in other indirect measures of social cognition. In response to a personal communication inquiring about implications of their findings for the desirability of using multiple sets of exemplars for IAT categories, along with multilevel modeling of the variance contributed by exemplars, Wolsiefer wrote that such use of exemplar sets and multilevel analysis “doesn’t appreciably change individual level bias scores . . . . [W]e also examined whether accounting for stimulus variance in the IAT would appreciably change the predictive validity of the IAT. We found no evidence that this was the case.” Even though it is often a desirable feature of research design, it does not appear necessary to develop multiple alternative sets of exemplars for target and attribute concepts in IAT measures. This proves fortunate because, in many cases, easy-to-classify exemplars are in short supply.

#### B7. It is desirable (not essential) for the number of trials in any block to allow each target exemplar stimulus to be presented the same number of times within the block, and likewise for the exemplars in each attribute category

The desirability of this practice is the usual desirability of minimizing sources of extraneous variance in data due to differences in procedures experienced by research subjects. Adoption of this practice can run into complications in managing equal appearances, due to the numbers of exemplars selected for each target category and each attribute category. To achieve equal appearances, within each combined-task block, of all attribute-concept or of all target-concept stimuli, trials in combined-task blocks must be twice the smallest value that is simultaneously an integer multiple of the number of unique target exemplars and unique attribute exemplars. For example, with four exemplars per target category (total = 8 exemplars) and five exemplars per attribute category (total = 10 exemplars), the smallest number that is an integer multiple of both 8 and 10 is 40, requiring a combined task block to have twice that number, or 80 trials, which may be an excessive block length for some subject populations. An acceptable alternative is to distribute the total of 80 trials across the two blocks of each combined task (an example is described in Appendix A). When equal numbers are not possible, it is generally easy to manage stimuli so that no exemplar of a target category is presented more than once more per block than any other exemplar of a target category (and similarly for attribute categories).

#### B8. Runs of more than four consecutive same-key-correct trials in combined-task blocks are undesirable

Runs of consecutive trials that require the same (left or right) key for a correct response allow subjects to increase their performance speed in the IAT due to a well-known repetition priming process (e.g., Horner & Henson, 2008) that is unrelated to strengths of associations between categories that share the same key. If these runs occur in one combined task and not in the other, they can inappropriately influence a subject’s IAT measure. And if they occur more for some subjects than others, they can similarly add statistical noise to estimates of means or correlations involving the IAT measure. Lengthy same-key-correct runs are avoidable in combined tasks by randomizing trials independently within each consecutive subset of four trials. Trials 1–4 would then randomly present a stimulus from one target concept on Trial 1 and from the other target concept on Trial 3, and a stimulus from one attribute concept on Trial 2 and the from the other attribute concept on Trial 4; and so on for Trials 5–8, 9–12, etc., with independent randomization for even-numbered and odd-numbered trials in each group of four trials. This strategy limits maximum same-key-correct runs to four trials. For comparison, randomization within groups of eight trials will allow occasional same-key-correct runs of eight trials (which is undesirable).

#### B9. In correlational studies, statistical power can be increased by using two (or more) administrations of the IAT for each subject

This strategy produces an IAT measure with greater test–retest reliability than is expected for a single IAT completion. (The statistical basis for this recommendation was described earlier in this article’s discussion of test–retest reliability, under the heading, Measurement Characteristics of IAT Measures.) Increased test–retest reliability will also reduce unsystematic variance in estimated sample means, providing both greater power in tests of experimental treatment effects and increased magnitude of correlations between IAT measures and conceptually related variables. An alternative means of gaining power for both of these purposes is to increase subject sample sizes. 9

#### B10. In studies that assess correlations of an IAT measure with other variables, it is desirable for the subject population to display substantial variability in the IAT measure

This expectation is a statistical consequence of the statistically necessary effect of restriction of range of a variable on the magnitude of correlations involving that variable (see, e.g., Cohen et al., 2003, p. 57). As example, if one assesses a correlation between gender identity (which varies widely between male and female) and gender attitude (which is correlated with gender identity), one observes a stronger correlation when the sample includes both male and female subjects than when the sample is either exclusively male or exclusively female. Similarly, a race attitude IAT (means of which vary substantially between African Americans and European Americans) will be more strongly correlated with a parallel self-report measure and with other theoretically connected measures when the research sample includes both racial groups than when the sample is limited to one of those two groups. This increased sensitivity to correlations is a justification for not subdividing a sample into demographically homogeneous groups when one or more variables being correlated differ non-trivially between the demographic groups that would thereby be analyzed separately.

#### B11. In laboratory research, when IAT-including experiments are administered by multiple experimenters, treatment conditions should be distributed equally across experimenters

This generally advisable research practice is recommended here because of its known importance in research using IAT measures. The effect of experimenter race on subject performance on race attitude IAT measures was first demonstrated by Lowery et al. (2001). Effects of other experimenter characteristics have not been established so clearly as for race of experimenter in the race attitude IAT, but are easily conceivable.

#### B12. Desirable procedures for pretest–posttest IAT administrations

The first IAT ever completed by a subject is known, on average, to show a more polarized result (i.e., greater distance from zero) than will a second or subsequent IAT completion (first reported by Greenwald, Nosek, & Banaji, 2003; see also Lai et al., 2016). This not-yet-fully-understood effect may be due to the first administration having slower responding on combined tasks than do subsequent administrations, if this slowing occurs more on the combined task that is more difficult for the subject. There are two ways to deal with the resulting expectation of a spurious change between the first and second IAT in a pre-post design: (1) Use a no-treatment control group that also receives both pretest and posttest (used first with IAT measures by Dasgupta & Greenwald, 2001), or (2) give all subjects pre-experimental IAT completion experience, which need not use the same IAT intended for the pretest–posttest design. Without one of these approaches, there is a risk of mistakenly interpreting an observed attenuation of IAT on a posttest as a treatment-caused reduction of the IAT.

### C. Recommended practices for reporting IAT procedures and results

It is often desirable to use IATs with procedures borrowed from a previous study that assessed the same or very similar constructs (i.e., attitudes, stereotypes, identities, or self-esteem). When portions of procedures in a new report are identical to ones reported in an accessible previous publication, it should suffice to cite the prior publication, giving the page number(s) on which those identically used procedures were described.

#### C1. Procedures that should be described in all empirical publications of original IAT results

##### Logistics and apparatus

Describe how subjects were recruited including any selection criteria or recruiting aids that were used. Report how many subjects began participation and causes of unavailability of data from those who began the procedure, including criteria for excluding partial or all data from participants who began the procedure. If conducted in a laboratory, the report should state laboratory location and physical properties, including nature of separation of multiple participants from one another; also computer type, operating system, monitor size, viewing distance, and laboratory software used to present IAT procedures and record data. If presented via Internet or otherwise remotely, describe software used for presentation and software or hardware required on the user’s side; also state whether procedures were presented full screen or otherwise.

##### Stimuli

Report all category labels and all exemplar stimuli (verbatim) for each category. If exemplars are words in a language other than English, many readers will appreciate having English translations (e.g., in parentheses). Report font name, size, and capitalization used for all categories with exemplars presented as words, and on-screen dimensions for pictures or graphics used for exemplars presented other than as words.

##### Trial presentation procedures

Give the numbers of trial blocks, the number of trials in each block, and indicate the categories assigned to left and right keys for each block. Either state that trials in combined-task blocks were strictly alternated between target and attribute categories (see Recommendation B3) or, if that was not done, describe the procedure for sequencing exemplars of the four categories. State whether procedures restricted runs of number of same-key-response trials in combined-task blocks (see Recommendation B8). Describe the intertrial interval within blocks of trials (i.e., the interval between initiating a key press and appearance of the next trial’s stimulus) and length of pauses between blocks of trials (or that pauses were ad lib, if they were under subjects’ control). Report (preferably verbatim) instructions provided to subjects about speed or accuracy of responding. Describe how erroneous responses were treated by the software and instructions (if any) given to subjects for what they should do on receiving error feedback. Describe counterbalancing (if any) for left or right side of correct responses for categories in Blocks 1 and 2 and the order of encountering combined-task blocks.

#### C2. Computation of IAT scores

The most widely used procedure for scoring IAT measures is the *D* score that was introduced by Greenwald et al. (2003; also described fully in this article’s Appendix B). The *D* algorithm affords a few scoring options for treatment of trials on which errors occurred; the error treatment method that was used should be reported. Although the *D* algorithm is resistant to disturbance by extreme scores, it does oblige exclusion of trials with latencies greater than 10 s and exclusion of entire data from subjects for whom more than 10% of trials have latencies faster than 300 ms. Subjects with this high a proportion of very fast responses are invariably ones who are trying to complete the procedure with maximum speed, pressing keys as rapidly as they can without concern for accuracy, and generally having error rates approximating 50%. Perhaps surprisingly, it is neither necessary nor desirable to drop subjects with relatively high error rates (even approaching 40%), so long as they are taking the procedure seriously and are trying to produce correct responses. The *D* algorithm does not truncate latency distributions to eliminate occasional very fast or very slow (short of 10 s) trials. If the scoring procedure in a report is deliberately varied from Appendix B’s recommendations, the variations and the justification for using them should be reported, to avoid readers’ concern about questionable research practices.

#### C3. Reporting of internal consistency of the IAT measure

Statistical measures of internal consistency estimate the degree to which multiple items in a self-report measure (such as a scholastic aptitude test or a self-esteem inventory) agree in what they are measuring. For an IAT measure, “items” are subsets of trials that suffice to compute an IAT measure. An IAT measure can be computed (very minimally) as a difference between latencies on two trials, one from each of the two combined tasks. If there are 60 trials in each combined task (as in the standard procedure described in Appendix A) it is possible to compute 60 IAT part-scores, which can be used for an internal consistency computation.

The extreme approach of computing two-trial IAT sub-measures is almost never used for internal consistency computations. Five more commonly used approaches are briefly described here, in order of increasing expected precision: (1) The computationally simplest approach is to correlate an IAT score computed with data from Blocks 3 and 6 of the standard procedure (see Appendix A) with a second score computed similarly from Blocks 4 and 7. This is convenient because these two-part scores are obtained in computing the standard IAT scoring algorithm’s *D* measure (see Appendix B). (2) One part-measure can be computed from all odd-numbered trials in the IAT’s combined-task blocks, and the other from all even-numbered trials. This may seem problematic because the IAT’s strict alternation of target and attribute exemplars (see Recommendation B3) results in one of these part measures using data exclusively from target category trials, and the other exclusively from attribute category trials. (3) A random selection of half of the trials from each combined task can be used as one part, with the remaining trials being the second part. (4) More cumbersome to compute, but also the method most likely to make the two parts as comparable as possible, is to sort all trials for each combined task into the four distinct categories of stimuli (i.e., those for the two target categories and the two attribute categories), then take either a random half from each of the four categories as one part with the remaining trials as the other part, or (5) compute one part by dividing each of the four categories within each combined task in half by a method that will assure that neither of the two halves over-represents trials occurring either early or late in the procedure. Although the last of these five procedures is expected to provide the greatest precision, all of the last three are quite satisfactory.

#### C4. Reporting of test–retest reliability of IAT measures

Test–retest reliability of IAT measures is computed as the correlation between two repetitions of the IAT measure obtained from the same sample of subjects. Because research studies rarely repeat the same IAT measure, test–retest reliability is very infrequently reported. Searching rigorously for reports of test–retest reliability of IAT measures, Greenwald and Lai (2000) found only 58 reports of test–retest reliability correlations. Perhaps this article’s Recommendation B9 will lead to more studies obtaining repeated IAT measures from the same subjects.

## Opportunities for improvement on current best practices

Since 1998, multiple attempts have been made to produce new latency-based indirect measures of the constructs assessed by IAT measures. Table 1 in Greenwald and Lai (2020) summarizes six such alternative methods. Additionally, there have been multiple proposals for alternative approaches to scoring the data produced by IAT measures (e.g., Conrey et al., 2005; Klauer et al., 2007) or by variants of IAT measures (e.g., Meissner & Rothermund, 2013). Although none of these alternatives is yet established as an improvement over the standard form of the IAT (see Appendix A) or the currently standard scoring algorithm (see Appendix B), there is no reason to conclude that continued or further efforts to improve IAT procedures and scoring methods will be futile. The following paragraphs describe three goals for potential improvement.

### Improving the statistical reliability of IAT measures

Increased test–retest reliability of IAT measures can benefit statistical power of all research designs (correlational or experimental) that use IAT measures. It can also enable (or at least advance) the possibility that IAT measures can become precise enough to accurately describe individual respondents’ levels of implicit bias. A method of improving test–retest reliability of any measure is to administer it to the same person multiple times, then average the two or more observed scores. As described earlier in this article, that strategy has been used very successfully with blood pressure measures in hypertension research. A necessary cost of the measure-repetition strategy is the time it adds to data collection. Because minimizing time is especially a priority in Internet-conducted research that can allow large samples, the measure-repetition strategy is more feasible for laboratory-administered studies than for Internet-administered studies. A more ambitious strategy for improving reliability is to find ways to reduce the statistical noise inherent in multi-trial reaction time measures. A successful noise-reduction strategy might well benefit from findings obtained with latency measures in areas of research other than social cognition. Because the problem of inherent noisiness of latency measures has been known for well over a half-century, the current lack of available solutions indicates how challenging this goal is.

### Extending the IAT to measure strengths of associations involving subcategories

“Subcategory” here refers to a concept defined by the intersection of two categories. Some such intersections that have already attracted interest are those of race with age (for example, Asian child as distinct from Asian adult) or race with gender (such as Black female as distinct from Black male or White female). Associations with such intersectional categories are conceivably either (a) the logical union of attributes associated with the two intersecting categories—i.e., the sum of attributes associated with the two categories, or (b) the logical intersection of attributes of the two categories—i.e., only those attributes that are associated with both categories. More interesting than both of those possibilities, but also more challenging, is that the intersection may be a qualitatively distinct category with its own associated attributes.

At present, no method exists for using an IAT to assess associations for an intersection of two demographic categories that may be different from associations with the categories individually. What might intuitively appear the obvious way to compare different intersections of two demographic categories in an IAT is to select, as exemplars for each intersection, persons who embody that intersection. For example, an IAT contrasting the intersection, Black female, with the intersection, Black male, might illustrate each with images—one set of images of Black women and one set of Black men. Pilot testing experience has revealed that subjects respond to such an IAT as if the images possessed only the attribute that distinguishes the two sets of images—in this case, only gender. Specifically, in an IAT with attribute categories of career vs. family, if the two target concepts are Black female and Black male, the IAT is expected to produce the same finding as an IAT involving all racially White or all racially Asian person images. The suspected reason for this (not yet strongly tested in experimental research) is that producing correct responses requires attending only to the attribute (gender, in these cases) that distinguishes the two sets of images. It is apparent that some other approach is needed if an IAT procedure is to be successfully adapted to assessing associations with category intersections.

### Extending the IAT to measure strengths of associations involving propositions

Several researchers have sought to construct IATs that could measure associations of agreement or disagreement with propositional meanings. One approach to this task was developed by Sartori et al. (2008), measuring association of truth or falsity with autobiographical events. In this task, categories of *true* and *false* were represented by exemplars in the form of brief sentences that could easily be classified as factually true or false. Others have subsequently similarly used propositions as exemplars for non-propositional categories (e.g., De Houwer et al., 2015; Lu, 2016). This appears to be a successful strategy, although it is not yet apparent how broadly it can be applied.

An alternate use of propositions in IAT measures is for the propositions to be category labels for the IAT, rather than category exemplars. One successful use of propositions as category labels for an IAT is the *personalized IAT* (Olson & Fazio, 2004) in which the propositional labels *I like* and *I don’t like* were used in place of the (non-propositional) *pleasant* and *unpleasant* attribute labels of most other IAT attitude measures. A second use of propositions as category labels is in the *wanting IAT*s developed by Koranyi et al. (2017) and by Tibboel et al. (2017). In these, category labels represent a goal-directed motivational state, using propositional category labels of *I want* and *I do not want*. Koranyi et al.’s procedure also required making subjects thirsty (by eating at least 8 salty crackers in two minutes) prior to completing the IAT.

Not yet attempted in propositional category labels are propositions with any complexity of syntactic structure or propositions with verbs other than ones expressing liking or wanting. An example of a conceivable IAT with greater complexity and other verbs in propositions is one in which the contrasted target concepts are *legalize abortion* and *outlaw abortion*. A challenge that has no obvious solution (for this hypothetical example) is to create exemplars that subjects can easily classify as representing these two contrasted propositions. Although recommended best practice B6 advises using at least three exemplars for an IAT category, it is conceivable that, with propositional target concepts, using the proposition itself as the sole exemplar might suffice. That may succeed if the contrasted propositions are only two words in length, but consider a more complex contrasted pair of propositions: *European countries should pay a larger fraction of their GDPs than the U.S. to cover NATO costs* and *U.S. and Europe should pay the same fraction of their GDPs to cover NATO costs*. Instructions might be used to allow abbreviating these as *NATO costs: EU more than US* and *NATO costs: EU same as US*. Either these or the pair involving abortion could be used in IATs to assess strength of association of these policies with contrasted political ideologies, political parties, or political candidates. Such research remains to be done.

## POSTSCRIPT: 29-person jury evaluates this article’s recommended practices

Writing of this article started in early 2018, a few months before the 20th anniversary of the IAT’s first publication. That seemed a good time to review what had been confidently learned from empirical research using the IAT as research method, as well as to consider the history of theoretical and methodological controversies concerning the IAT, and also to summarize current wisdom on best practices for conducting research using IAT measures. Overviews of accumulating knowledge and discussions of interpretive controversies had been treated in several handbook chapters and journal review articles during the last decade (see the overview of those reviews by Greenwald & Lai, 2020). However, best practices for research using IAT measures had not received any published treatment since two book chapters published in 2007 (Lane et al., 2007; Nosek et al., 2007). The first author’s initial attempt at a new summary of best research practices produced Draft 1 of a manuscript titled “The Implicit Association Test at Age 20: What is known and what is not known about implicit bias” (“Age20” hereafter).

Draft 1 of Age20 was circulated to 73 researchers and scholars who were (a) authors of empirical or theoretical articles concerning the IAT or (b) among the 32 participants at a September 2017 National Science Foundation “Implicit Bias Conference” or (c) among the 26 presenters at an upcoming November 2018 Ghent University conference titled “Improving the Utility of Indirect Measures”. These three overlapping lists included researchers with widely ranging views on understanding of the IAT as a psychological measure. The message to each of these 73 described the manuscript’s purpose as “to describe a consensus on what is currently known about the IAT”, adding: “The attached draft includes [the first author’s] views on what is known—hardly a consensus—[and seeks] opinions and suggestions to convert this version into one with reasonable claim to reporting a consensus.”

Within a month after Draft 1’s circulation, 25 of the invitees had responded. Sixteen of them provided extensive comments, each prompting follow-up exchanges of email that provided the bases for multiple revisions. In November 2018, the Ghent University conference provided opportunity not only for discussions of issues raised by several of the attendees in response to Draft 1, but also allowed discovery of additional well-qualified researchers among the conference’s non-presenting attendees. In late November of 2018, Draft 2 was circulated to the original 73 plus 14 more. Four of the new recipients sent substantial comments on Draft 2, as did several of the previous commenters, leading to further revisions.

Although the responses to Drafts 1 and 2 prompted useful revisions, they did not allow assessment of consensus for the various research practice recommendations. To overcome that limitation, a spreadsheet that could assess consensus was prepared. The spreadsheet gave each recipient the role of a juror who was asked to judge *agree*, *disagree*, or *uncertain* for each conclusion statement. The instruction on the spreadsheet concluded, “Please be reluctant to use the uncertain response when you are aware of a balance of evidence favoring agree or disagree. Feel free to add a comment in the NOTES column if it might be useful, but treat this as optional.” Draft 3, accompanied by the spreadsheet, was circulated in late December, 2018 to all those who had received Draft 2. Twenty-five spreadsheets were returned. All of Draft 3’s conclusions received majority agreements on the spreadsheet, but there were nevertheless multiple judgments of disagreement with individual recommendations, mostly accompanied by explanations of the bases for disagreement. Further follow-up correspondence made clear that common ground would not be found for a few of the conclusions, warranting one further draft.

Draft 4 was circulated in March, 2019 to the 35 commenters who had provided comments (most more than once) on the three previous drafts. Draft 4 was accompanied by a revised spreadsheet, which stated the recommended practices very similarly to their appearance in this article.^10^ Two of Draft 1’s eleven recommendations for selection of categories and selection of exemplar stimuli were dropped because of insufficient consensus. For four of the 12 recommendations for administering IAT measures, Draft 4 included revised content that addressed concerns that various commenters had expressed in response to earlier drafts. Circulation of Draft 4 was accompanied by messages to each of the 35 commenters, including (as appropriate) description of how Draft 4 had been revised in response to their latest comments, and also requesting their use of an accompanying repeat copy of the revised spreadsheet to revise any judgments that warranted change in consideration of Draft 4’s revisions that had attempted to accommodate the final wave of comments. For the ten who had not previously provided spreadsheets, there was a reminder that a completed spreadsheet would still be very welcome. In response to that invitation, four new spreadsheets were received, in addition to five from previous providers of spreadsheets that contained one or more revised judgments.

The just-reviewed process of preparing Age20 yielded 29 spreadsheets from authors and commenters, each providing evaluations of agree, disagree, or uncertain for each of Table 1’s recommendations.^11^ The original 73 invitees could be classified into (a) persons who were known, on the basis of their past publications, to be favorable to the IAT as a research procedure (*N* = 25 [34%]), (b) persons known to be critics of the IAT in one respect or another (*N* = 18 [25%]), (c) persons ambivalent regarding the IAT, meaning that their publications used IAT measures but sometimes reached IAT-critical conclusions (*N* = 10 [14%]), and (d) persons who were knowledgeable about the IAT but who had done neither supporting nor critical research (*N* = 20 [27%]). For the final 29 who became the 29 judges, the corresponding numbers in those four categories were (a) 19, (b) 3, (c) 5, and (d) 2. Considering that the IAT-favorable invitees had the greatest research experience using IAT measures, it is not surprising that about 2/3 of the 29 (compared to only about 1/3 of the full set of invitees) were in that category.

For the final judgments of this article’s recommended research practices, 3.9% of all judgments were in the “disagree” category and 9.4% were “uncertain”. The remainder (86.7%) were judgments of “agree”. Understandably, most of the “uncertain” judgments were for recommendations that had been based only on researchers’ pilot testing experience. Only two best practice recommendations received more than 2 “disagree” judgments from the 29 judges. These were A7, which was the recommendation to avoid attribute exemplars that had some extraneous basis for association with one of the two target concepts, and B1, the recommendation to routinely counterbalance order of combined tasks. The bases for these “disagree” judgments are described in this article’s sections that present these recommendations.

For B2–B12, there were more than two judgments of uncertainty for six of the recommended best practices. Greatest uncertainty (*N* = 9) was expressed for Recommendation B5, that initial practice in classifying the two target concepts should precede initial practice in classifying the two attribute concepts. Next most uncertainty (*N* = 7) was for Recommendation B3, to strictly alternate target and attribute trials in combined tasks. The uncertainty in these two cases and the lesser uncertainty for a few other recommendations are understandable given that the primary basis for each of these recommendations was unpublished pilot testing experience of previous researchers rather than controlled experiments. For all of B2–B12, the judgments of more than 90% of those who expressed either agreement or disagreement were judgments of agreement. Even though further research to evaluate some of these recommendations is clearly desirable, the high levels of expressed agreement justified regarding these as recommended practices until further empirical evidence indicates otherwise.

## Appendix A “Standard” (seven-block) IAT procedure

As most frequently used in research, an IAT consists of seven sets (blocks) of trials in which stimuli from four categories are classified. Any IAT is completely specified by the labels to be used for the four categories and the stimulus items (exemplars) used to represent each of the four categories. The subject’s task in each of the seven blocks is to provide correct classifications of stimulus items by pressing an assigned left- or right-positioned key on a computer keyboard—for example “E” and “I” (alternately, “D” and “K”) on a QWERTY keyboard—into their categories. Most often, two of the categories are identified as *target* categories. The first reported IAT (Experiment 1 in Greenwald, McGhee, & Schwartz, 1998) used *flower* and *insect* as the labels for its two target categories. The other two categories are typically identified as *attribute* categories. In the flower–insect attitude IAT the attribute categories were *pleasant* and *unpleasant* (valence).

The standard order of seven blocks (typical trial numbers [totaling 190] in parentheses), is

1. Classify the items for the two target categories (20)
2. Classify the items for the two attribute categories (20)
3. Classify items for all four categories, one attribute and one target category assigned to each of the two keys, using the assignment of categories to left and right keys as in Blocks 1 and 2 (20).
4. Same as Block 3 (40).^12^
5. Classify the two target categories, reversing the key assignments of Block 1 and having more trials than in Block 1 (30).
6. Classify items for all four categories, using the reversed key assignments of the target categories as in Block 5 (20).
7. Same as Block 6 (40).

The number of trials for reversed two-category practice in Block 5 can affect the magnitude of effect on the IAT of the order in which the two combined tasks are encountered. After several years of experience, an increase from 20 to 30 trials in Block 5 was adopted as a procedure that often keeps the effect of order of combined tasks to a minimum.

For the four combined-task blocks (3, 4, 6, and 7), which present exemplar items from all four categories, there is a *strict alternation* between presenting an item from one of the two target categories on odd-numbered trials and an item from one of the two attribute categories on even-numbered trials (see B3). Determination of which target category is assigned a left (vs. right) key response in Block 1 and how attribute categories are assigned to keys in Block 2 are typically counterbalanced across subjects. There are typically between four and six items in each of the four categories. The number of trials in a block is often adjusted to allow each of the stimuli to appear equally often. With the same number of exemplars (*n*) for each of the four categories, this can be done in the two-category blocks (1, 2, and 5) by having trial counts that are integer multiples of 2*n*, and in the combined-task blocks (3, 4, 6, and 7) trial counts being an integer multiple of 4*n*. With five items per category, the numbers might be as shown in the seven-block listing above. With four items per category, the numbers of trials in the seven blocks might by 16, 16, 32, 48, 24, 32, 48. For six items per category, these numbers might be 12, 12, 24, 48, 24, 24, 48.

As stated in Recommendations B6 and B7, however, exactly equating numbers of presentations for target or attribute exemplars should be subordinated to other considerations in determining the trial count for each block. As one example, the numbers for four items per category might be set at 16, 16, 24, 40, 24, 24, 40. The number of appearances of each item in combined tasks can then be equated because the sum of trials in each combined-task’s pair of blocks is an integer multiple of 4*n*—e.g., for Blocks 3 and 4 the sum is 24+40 = 64 (= 4 × 4*n*). Other numbers of items per category, especially with different numbers of exemplars in attribute and target categories, might require inappropriately large numbers of trials to maintain equal appearances of each exemplar for target and/or attribute categories. The strict equality need not be treated as essential.

A procedure that records latency to occurrence of the correct response is typically used, with the IAT program recording occurrence of error responses but not registering the trial’s latency as completed until the correct response occurs. The value of this *built-in-error-penalty* method was shown by Greenwald, Nosek, and Banaji (2003). For laboratory software that does not permit waiting for occurrence of the correct response on each trial, a scoring method with latency penalties in the form of milliseconds added to error trials is described in Appendix B.

## Appendix B

**Table:**

**Algorithms for the IAT’s** ***D*** **measure**		
**Step**	**Built-in error penalty procedure (preferred)** *Each trial’s latency is recorded to occurrence of the trial’s correct response; trials on which errors preceded the correct responses are included*	**Computed error penalty** *For IAT procedures that end a trial on the first keypress, recording the latency of that keypress and code the response as correct or error*
1	Designate combined tasks as A (for which faster performance will produce a positive score) and B (for which faster performance will produce a negative score). With counterbalancing, half of subjects will encounter A in Blocks 3 & 4, half in Blocks 6 & 7	Same
2	Discard all trials in Blocks 1, 2, and 5	Same
3	Identify blocks for combined task A as A1 and A2; those for combined task B as B1 and B2. If task A is Blocks 3 & 4, Block 3 is A1, Block 4 is A2	Same
4	Eliminate from remaining data (Blocks 3, 4, 6, and 7) *only* trials with latencies > 10,000 ms	Same
5	Eliminate all subjects for whom *more than* 10% of remaining trials have latencies faster than 300 ms	Same
6	Compute latency means (MnA1, MnA2, MnB1, MnB2) and SDs (SDA1, SDA2, SDB1, SDB2) for each of the four blocks for all remaining trials	Compute latency means for *correct responses* in each of the four blocks (separately) for remaining trials; also, replace each error response with a score computed as the *mean of correct responses in the same block as the error,* *plus a penalty* (see the note below this table)
7	Compute two mean latency differences: B1–A1 = (MnB1 – MnA1) and B2–A2 = (MnB2 – MnA2)	Compute the two mean latency differences from all trials, including the error trials that were replaced in Step 6 using error penalties
8	Compute an *inclusive* (not pooled) SD1 using all latencies in Blocks A1 & B1; another (SD2) using all latencies for A2 & B2 (SD2). These can be computed from means and SDs from Step 6 as shown in the lines below this table	Compute the two inclusive SDs using all trials (using the error trials with their replaced latencies)
9	Compute (B1–A1) / SD1; and (B2–A2) / SD2	Same
10	*D =* Average of two quotients computed in Step 9	Same

$$ {\displaystyle \begin{array}{c}\mathrm{SD}1=\mathrm{SQRT}\left(\left(\left(\left(\mathrm{NA}1-1\right)\ast \mathrm{SDA}1\hat{\mkern6mu} 2+\left(\mathrm{NB}1-1\right)\ast \mathrm{SDB}1\hat{\mkern6mu} 2\right)+\left(\left(\mathrm{NA}1+\mathrm{NB}1\right)\ast \left(\left(\mathrm{MnA}1-\mathrm{MnB}1\right)\hat{\mkern6mu} 2\right)/4\right)\right)/\left(\mathrm{NA}1+\mathrm{NB}1-1\right)\right)\\ {}\mathrm{SD}2=\mathrm{SQRT}\left(\left(\Big(\left(\mathrm{NA}2-1\right)\ast \mathrm{SDA}2\hat{\mkern6mu} 2+\left(\mathrm{NB}2-1\right)\ast \mathrm{SDB}2\hat{\mkern6mu} 2\right)+\left(\left(\mathrm{NA}2+\mathrm{NB}2\right)\ast \left(\left(\mathrm{MnA}2-\mathrm{MnB}2\right)\hat{\mkern6mu} 2\right)/4\Big)\right)/\left(\mathrm{NA}2+\mathrm{NB}2-1\right)\right)\end{array}} $$

In the above two lines, ‘N’, ‘Mn’, and ‘SD’ indicate numbers of trials, means, and standard deviations for the block indicated by the following two characters (A1, B1, A2, or B2); the caret (^) precedes an exponent.

Table 2 of Greenwald, Nosek, & Banaji (2003) suggested two options for the error penalty computation. One of these (*D*_*3*_) used twice the block’s SD (i.e., twice SDA1, SDA2, SDB1, or SDB2, depending on the block in which the error occurred). The other option (*D*_*4*_) used a constant of 600 ms for all blocks. Greenwald et al. also noted the option of deleting responses faster than 400 ms, a procedure that typically affects the resulting measure very little.

## Footnotes

1 In late June of 2021, the American Psychological Association’s PsycNET database contained 4459 publications that included “Implicit Association T*” in at least one of the fields of Title, Abstract, Keywords, or Tests and Measures. The retrieved items included 3363 peer-reviewed journal articles and 238 dissertation abstracts. This count does not include numerous publications in disciplines outside of psychology, including medicine, law, political science, business, education, and economics.

2 Greenwald and Banaji (1995) defined implicit social–cognitive constructs as “introspectively unidentified (or inaccurately identified) traces of past experience that mediate [response to] social objects” (p. 8). That definition remains useful, but is silent on the measurement method vs. mental representation/process distinction that has been focal in subsequent treatments of the definition of ‘implicit’, such as those of Fazio and Olson (2003) and De Houwer et al. (2009a, 2009b)

3 The direction of scoring of IAT measures is arbitrary, established by the researcher’s choice of direction in which the difference between mean latencies in the IAT’s two combined tasks (see Appendix A) is computed (see Appendix B).

4 The intercept in units of the *D* measure was not reported by Cvencek et al. (2020). The figure reported here required a computation that reversed the direction of the regression reported by Cvencek et al.

5 Limited test–retest reliability is typical for latency-difference measures such as the IAT. Draheim et al. (2019) reviewed the challenges posed by limited reliability of latency-difference measures that are used in cognitive psychological research.

6 Correlations of the order of administering combined tasks with IAT measures ranged from – .02 to .25 in data reported by Greenwald et al. (2003). For an observed correlation of *r* = .40 between the IAT and another variable of interest, using order of tasks as a covariate and assuming the largest of previously observed order-effect correlations (.25) would increase that correlation by only .013, from .400 to .413. (Still, it would be appropriate to conduct this covariance analysis and report the (slightly) larger correlation as the observed correlation.)

7 This possible effect depends on an assumption that a *right side=positive* cultural association can inflate positivity of a positively valenced concept when the right key is associated with positive valence in Blocks 2, 3, 4, 6, and 7 of the standard IAT.

8 Paivio’s analysis almost certainly also explains why evaluative priming experiments generally use the concept categories (racial groups, ethnic groups, gender groups) as primes, rather than using the attribute categories as primes (e.g., Fazio et al., 1986).

9 A reviewer of the original submission of this article expressed concern about administering multiple IATs to research subjects, suspecting that subjects with experience taking IAT measures are likely to try to fake IATs. This seems a needless concern. There appears to be no reason for concern that subjects more than very rarely approach a research study with an intent to fake their IAT responses. Rather, research subjects generally appear to be focused on producing correct responses in the IAT. Greenwald et al. (2020, pp. 22, 24) reviewed findings showing that subjects not instructed in how to fake an IAT typically are unable to deploy an effective faking strategy. In research studies that are not explicitly studying IAT faking, IATs are generally administered with no instructions designed to head off faking. Of multiple studies involving subjects instructed to fake (but without instructions in how to fake effectively) only Röhner et al. (2011) found statistically significant IAT faking. That finding was limited to one of the two directions of instructed faking; the authors did not report a combined statistical test including both directions of instructed faking.

10 A few had subsequent rewordings that did not alter their meaning, including B10, which was changed from a recommendation of what not to do to a recommendation of what to do.

11 The 29 judges sometimes left blank responses, which were treated as equivalent to a judgment of “uncertain”.
